# FZC-TDE: The Algorithm for Real-Time Ultrasonic Stress Measurement at Low Sampling Rates

**DOI:** 10.3390/mi16121340

**Published:** 2025-11-27

**Authors:** Feifei Qiu, Bing Chen, Chunlang Luo, Jiakai Chen, Ziyong He, Jun Zhao, Guoqing Gou

**Affiliations:** 1 School of Materials Science and Engineering, Southwest Jiaotong University, Chengdu 610031, China; qffswjtu@163.com (F.Q.); chenbing@my.swjtu.edu.cn (B.C.); luochunlang525@my.swjtu.edu.cn (C.L.); jiakia_chen@163.com (J.C.); 2 Dongfang Electric Machinery Co., Ltd. Deyang 618000, China; hezy6887@dongfang.com (Z.H.); runner_wxlz@163.com (J.Z.)

**Keywords:** ultrasonic stress measurement, low sampling rates, time delay estimation (TDE), frequency-domain zero padding, cross-correlation algorithm

## Abstract

Micro–nano-sized processing equipment requires high levels of precision, necessitating residual stress measurement to maintain stability. Ultrasonic stress measurement is an effective method but is hindered by high sampling-rate requirements, leading to excessive power consumption and hardware costs. This study presents a low-sampling-rate method based on the novel Frequency-domain Zero-padded Cross-correlation Time Delay Estimation (FZC-TDE) algorithm. Tensile validation experiments determined the minimum hardware sampling-rate requirement: rates below 25 MSps (even with interpolation) fail to characterize temporal delay variations effectively, and a rate of at least 20 times the signal frequency is required for ±10 MPa accuracy. The proposed FZC-TDE utilizes a frequency-domain fusion operation (frequency-domain zero-padding interpolation combined with cross-correlation) to enable real-time, high-resolution delay measurement at low rates. Comparative experiments show that time-domain interpolation methods (Linear, PCH, Cubic Spline) achieve similar stress estimation accuracy at the same rate (e.g., 7.4–8.7 MPa error at 100 MSps), while FZC-TDE (10.3 MPa error) offers superior computational efficiency. At 100 MSps, FZC-TDE maintains a stable computation time (~2.8 ms), while those of interpolation methods increase significantly (20–30 ms) due to higher oversampling factors. Furthermore, FZC-TDE reduces the number of arithmetic operations by 75% (2.26 million vs. ≥9.18 million for 128× oversampling on 1024 points) and exhibits slower computational load growth with oversampling ratios. Thus, FZC-TDE provides an optimal balance of acceptable accuracy and significantly enhanced efficiency, particularly for real-time or resource-constrained applications. This work reduces sampling-rate constraints and supports advancements in micro–nano-sized processing equipment and device performance.

## 1. Introduction

The utilization of micro–nano-sized processing equipment, such as lithography and etching machines, requires precise operation. The requirements for equipment component stability, including workpiece tables, mask tables, and load-bearing tables, exceed those in conventional industrial domains, such as the automotive, bridge, and railway industries [1,2,3]. However, during the manufacturing of micro–nano-sized processing system components, plastic deformation and heat input cause residual stress to build up inside components [4,5]. During the long service life of these components, stress may be released due to aging, causing changes in their dimensions and thereby affecting system accuracy. Therefore, the residual stress testing of micro–nano-sized processing equipment components to assess their internal stress distribution is a key measure for ensuring stable equipment operation.

Non-destructive stress testing methods include the X-ray diffraction method [6,7], the magnetic method [8], and the ultrasonic method [9]. X-ray diffraction technology is limited by its shallow penetration depth (10–30 μm) and low measurement efficiency, making it unsuitable for large-area stress detection [10]. Magnetic measurement methods can only be used to assess the stress of ferromagnetic materials, and therefore are unsuitable for testing stress in non-ferromagnetic materials, such as aluminum alloys, ceramics, etc. The Longitudinal Critically Refracted (LCR) wave method is a commonly used ultrasonic stress measurement technique, with the advantages of wide material applicability, fast measurement speed, and low cost, making it a promising stress assessment method.

Ultrasonic propagation time difference, also known as time delay, is an important parameter in LCR wave stress measurement which reflects the speed of sound in a medium. As there is a specific correlation between sound speed and stress, the accuracy of time delay measurements directly affects the accuracy, resolution, and stability of stress measurements. Time delay estimation algorithms are primarily classified as either feature point methods or waveform similarity methods. In the former, the propagation time is calculated using the waveform feature points, such as zero-crossing points [11] and peak points [12], of two signals, followed by time delay calculation. Methods of this type are associated with a relatively fast calculation speed; however, they can only be used for signals with a high signal-to-noise ratio, and Time Delay Estimation (TDE) is easily affected by thresholds or signal noise. Improving the accuracy of feature point methods typically requires signal processing techniques such as signal reconstruction [13], high-order filtering [14], and least squares fitting [15], which can indirectly increase the computational load and reduce efficiency.

The cross-correlation algorithm [16,17,18], one of the most widely used time delay estimation algorithms, estimates time delays by comparing the degree of correlation between two discrete sequence signals. This algorithm can achieve high measurement accuracy even under low signal-to-noise ratio conditions [19,20]. Improving the time resolution of delay estimation without increasing the hardware sampling rate often involves using interpolation algorithms to perform operations on the signal. The multi-channel cross correlation coefficient (MCCC) has been proposed based on linear interpolation and cross correlation, with research showing that it has good resistance to reverberation and noise. Guschina et al. [21] observed polynomial interpolation methods applied to a sampled cross-correlation function in the neighborhood of its maximum, and their research shows that the time delay estimation values are nearly identical for the absolute and conjugate approximation methods, differing only in computational efficiency. Sun et al. [22] proposed the principle of time-of-flight measurement based on the cross-correlation method, introduced Cubic Spline interpolation, and carried out experimental analysis. In their study, the signal was first interpolated, and then cross-correlation was used to estimate the time delay.

Current ultrasonic stress measurement systems use a hardware sampling rate of 2.5 GSPs to achieve a time resolution of 0.1 ns [23,24]. However, this high sampling rate leads to high hardware costs [25]. Although reducing the sampling rate can reduce hardware costs, the temporal resolution at low sampling rates is often insufficient to meet the demands of rapid measurement. Therefore, there is an urgent need for an algorithm that achieves both time delay estimation accuracy and computational efficiency. Furthermore, the impact of the sampling rate on the accuracy of ultrasonic stress measurement has not yet been thoroughly studied.

In this study, we conducted tensile validation experiments at different sampling rates to determine the minimum hardware sampling-rate requirements for ultrasonic stress measurements. Then, frequency-domain zero-padding interpolation and cross-correlation time delay estimation were analyzed in detail, and a novel algorithm, Frequency-domain Zero-padded Cross-correlation Time Delay Estimation (FZC-TDE), was developed. In order to verify the performance of the FZC-TDE algorithm, comparative experiments were carried out.In this study, we conducted tensile validation experiments at different sampling rates to determine the minimum hardware sampling-rate requirements for ultrasonic stress measurements. Then, frequency-domain zero-padding interpolation and cross-correlation time delay estimation were analyzed in detail, and a novel algorithm, Frequency-domain Zero-padded Cross-correlation Time Delay Estimation (FZC-TDE), was developed. In order to verify the performance of the FZC-TDE algorithm, comparative experiments were carried out.In this study, we conducted tensile validation experiments at different sampling rates to determine the minimum hardware sampling-rate requirements for ultrasonic stress measurements. Then, frequency-domain zero-padding interpolation and cross-correlation time delay estimation were analyzed in detail, and a novel algorithm, Frequency-domain Zero-padded Cross-correlation Time Delay Estimation (FZC-TDE), was developed. In order to verify the performance of the FZC-TDE algorithm, comparative experiments were carried out.

## 2. Measurement Theory

### 2.1. Theoretical Considerations Regarding LCR Wave Stress Measurement

**Figure 1:**
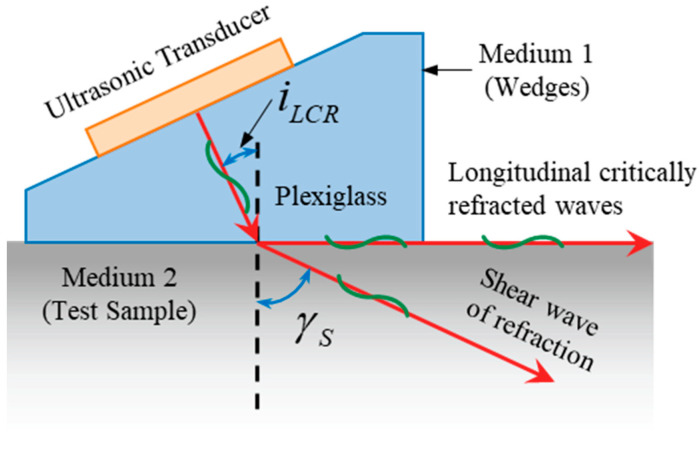
Sketch showing LCR-wave propagation.

An LCR wave propagates parallel to the surface at a certain depth, as shown in Figure 1. The first critical angle can be calculated with the following equation:

(1) $$i_{LCR}=\sin^{-1}(V_{1}/V_{2})$$

where *V*_1_ and *V*_2_ are the propagating velocities in media 1 and 2, respectively; *i_LCR_* is the first critical angle; and γS is the shear angle of refraction.

Within the elastic limit, the ultrasonic stress measurement technique relies on the linear relationship between stress and the travel time of the sound wave, i.e., the acoustoelastic effect. The relationship between stress and the velocity of the longitudinal plane waves traveling parallel to the load is shown in the following equation [26]:

(2) $$\rho_{0}V_{11}^{2}=\lambda +2\mu +\frac{\sigma }{3\lambda +2\mu }\left[\frac{\lambda +\mu }{\mu }\left(4\lambda +10\mu +4m\right)+\lambda +2l\right]$$

where *V*_11_ is the velocity of the waves in the direction of medium 1 with particle displacement in the same direction; ρ0 is the initial density of the material without stress; λ and μ are the Lame constants; l, m, and n are the Murnaghan constants; and σ is the stress.

In the natural state, the sound velocity of the sample can be expressed as

(3) $$V_{0}=\sqrt{\frac{\lambda +2\mu }{\rho_{0}}}$$

Substituting Equation (3) into Equation (2) yields

(4) $$V^{2}=V_{0}^{2}(k_{1}\sigma +1)$$

where k1 is the acoustoelastic coefficient, because the Lame and Murnaghan constants of the body hardly change under elastic conditions. Therefore, k1 is approximated by a constant according to the following equation:

(5) $$k_{1}=\frac{\frac{4\lambda +10\mu +4m}{\mu }+\frac{2l-3\lambda -10\mu -4m}{\lambda +2\mu }}{3\lambda +2\mu }$$

In Equation (4), taking the derivative of V with respect to stress σ yields

(6) $$\frac{V}{V_{0}^{2}}\frac{dV}{d\sigma }=\frac{k_{1}}{2}$$

Stress-induced changes in ultrasonic wave velocity are minimal at low stress levels, supporting the approximation V/V0 ≈ 1. Equation (6) can then be simplified to

(7) $$d\sigma =\frac{2}{k_{1}V_{0}}dV$$

Assuming that the acoustic path length of the LCR wave in the workpiece is L and its propagation time is t, the derivative of its velocity V with respect to t is expressed as

(8) $$dV=-\frac{L}{t^{2}}dt$$

We substitute Equation (8) into Equation (7):

(9) $$d\sigma =-\frac{2L}{k_{1}V_{0}t^{2}}dt$$

Since the acoustoelastic effect is negligible, t≈t0 can be approximated, and Equation (9) can be further simplified to

(10) dσ=K⋅dt

where K is the stress coefficient of the workpiece, expressed as

(11) $$K=\frac{-2V_{0}\left(3\lambda +2\mu \right)}{\left(\frac{4\lambda +10\mu +4m}{\mu }+\frac{2l-3\lambda -10\mu -4m}{\lambda +2\mu }\right)L}$$

The relationship between stress and acoustic time difference can be obtained with Equation (10).

### 2.2. FZC-TDE Methodology

#### 2.2.1. The Direct Cross-Correlation Method

Cross-correlation time delay estimation algorithms are mainly divided into direct cross-correlation and improved versions of the cross-correlation algorithm, including the generalized cross-correlation algorithm [18], the Hilbert transform [27], weighted cross-correlation [28], and phase-corrected cross-correlation [29]. Although these improved algorithms have better noise adaptability, they increase the number of operational steps due to direct cross-correlation, which reduces the computational efficiency of the algorithm. Therefore, the direct cross-correlation algorithm, with minimum computational complexity, was selected in this study. The cross-correlation function, *R*(*x*, *y*), is a measure of the degree of correlation between two signals (*x* and *y*). Moreover, *R*(*x*, *y*) can be used to determine the similarity of two waveform sequences [30]. The discrete time-domain cross-correlation operation is given by

(12) $$R(x,y)[k]=\sum_{i=-\infty }^{+\infty }x[i-k]y[i]$$

As shown in Equation (12), it can be seen that discrete cross-correlation is the deconvolution of the original signal followed by discrete convolution. Since time-domain convolution corresponds to frequency-domain multiplication, time-domain deconvolution equivalently becomes frequency-domain conjugation. Frequency-domain cross-correlation is therefore equivalent to the product of two spectral sequences, with the second signal’s spectrum being represented by its complex conjugate. The frequency-domain representation of the cross-correlation function is given by

(13) $$R(x,y)=IDFT\left\{DFT{\left\{x\right\}}^{*}\circ DFT\left\{y\right\}\right\}$$

**Figure 2:**
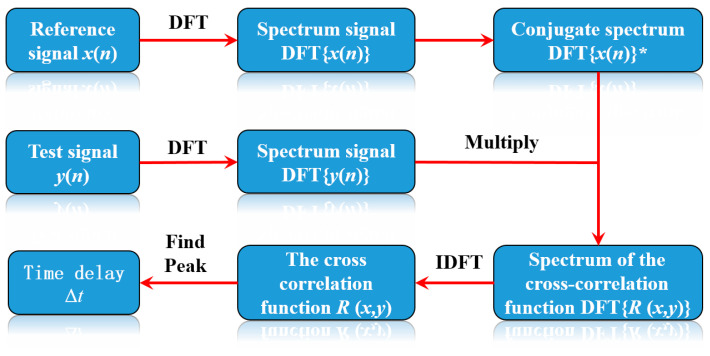
Flowchart of the cross-correlation time delay estimation algorithm.

The time delay can be estimated by determining the peak location of the cross-correlation function (*R*(*x*, *y*)) [31]. Signals *x*(*n*) and *y*(*n*) are converted into spectrum signals DFT{*x*(*n*)} and DFT{*y*(*n*)} with the DFT. The complex conjugate operation is then performed on DFT{*x*(*n*)} to obtain DFT{*x*(*n*)}*. DFT{*x*(*n*)}* and DFT{*y*(*n*)} are then multiplied to obtain spectrum DFT{*R*(*x*, *y*)} of the cross-correlation function. The inverse Fourier transform is then applied to DFT{*R*(*x*, *y*)} to obtain the cross-correlation function, *R*(*x*, *y*). The time delay estimate, Δ*t*, is obtained by determining the position of the maximum peak in *R*(*x*, *y*). Figure 2 illustrates the flowchart of the cross-correlation time delay estimation algorithm.

The temporal resolution of cross-correlation time delay estimation is fundamentally limited by the hardware sampling rate. To achieve enhanced temporal resolution without increasing the latter, the original signal needs to be interpolated.

#### 2.2.2. The Frequency-Domain Zero-Padding Interpolation Method

Commonly used interpolation algorithms include Frequency-domain Zero-padding (FZ) [32], Linear (Line) [33], Piecewise Cubic Hermite (PCH) [34], and Cubic Spline (CS) interpolation [35]. Compared with other algorithms, FZ interpolation has a low computational speed and complexity, and it operates in the frequency domain. The characteristics of frequency-domain operation mean that FZ interpolation can be fused with CCTDE in the frequency domain. Therefore, FZ interpolation was chosen in this study.

Consider a real-valued sequence *x*(*n*), where *n*∈[0, *N* − 1], with its corresponding discrete Fourier transform (DFT) being denoted by *X*_1_(*i*), where *X*_1_(*i*) = DFT{*x*(*n*)}, with *i*∈[0, *N* − 1]. The frequency-domain data *X*_1_(*i*) are then expanded by *M* times by applying zero padding in the middle region, resulting in an extended spectrum *X*_2_(*i’*), with *i’*∈[0, *M* × *N* − 1].

(14) $$X_{2}(i^{\prime })=\left\{\begin{array}{l} X_{1}(i),\;\;\;\;\;\;\;\;\;\;\;\;\;\;\;\;\;\;\;\;\;\;\;\;\;\;\;\;\;\;\;\;\;\;\;\;\;\;\;\;\;\;\;\;\;\;\;\;\;\;\;\;\;\;\;\;\;\;\;\;\;\;\;\;0\leq i^{\prime }\leq N/2\\ X_{1}(i^{\prime }-MN+N),\;\;\;\;MN-N/2\leq i^{\prime }\leq MN-1\\ 0,\;\;\;\;\;\;\;\;\;\;\;\;\;\;\;\;\;\;\;\;\;\;\;\;\;\;\;\;\;\;\;\;\;\;\;\;\;\;\;\;\;\;\;\;\;\;\;\;\;\;\;\;\;\;\;\;\;\;\;\;\;\;\;\;\;\;\;\;\;\;\;\;\;\;others \end{array}\right.$$

Time-domain sequence *x*_1_(*s*) is obtained by computing the inverse discrete Fourier transform (IDFT) of *X*_2_(*i’*), where *x*_1_(*s*) = IDFT{*X*_2_(*i’*)}, with s∈[0, *M* × *N* − 1]. *x*_1_(*s*) can be rewritten by leveraging the conjugate-symmetric property of the DFT as follows [32]:

(15) $$x_{1}(s)=\frac{1}{MN+1}\sum_{n=0}^{N-1}x_{1}(n)\left\{1+2\sum_{j=1}^{N/2}\cos\left(\frac{2\pi }{N}j\left(\frac{s}{M}-n\right)\right)\right\}$$

**Figure 3:**
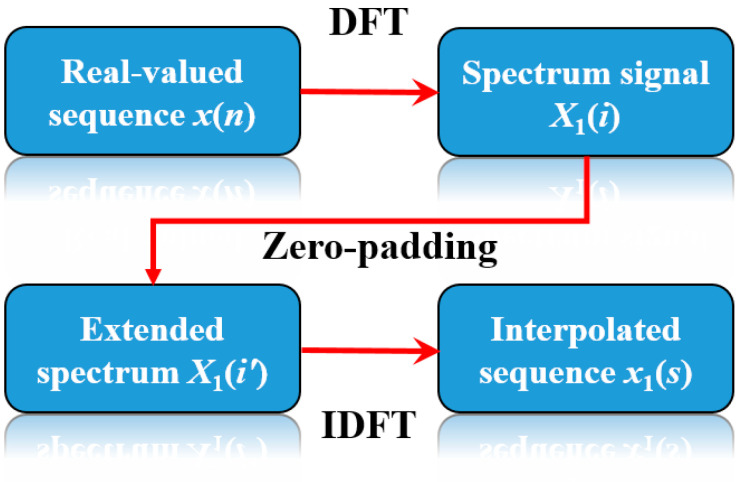
Flowchart of the frequency-domain zero-padding interpolation method.

Equation (15) describes a time-domain convolution of the original sequence with a coefficient sequence and shows that *x*_1_(*s*) is the time sequence after interpolating *x*(*n*) M times. The flowchart of the frequency-domain zero-padding interpolation method is shown in Figure 3.

#### 2.2.3. FZC-TDE Algorithm

**Figure 4:**
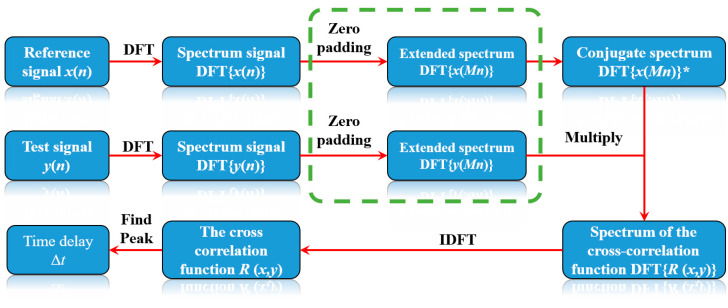
Flowchart of FZC-TDE.

Both frequency-domain zero-padding interpolation and cross-correlation-based time delay estimation, as demonstrated in Figure 2 and Figure 3, inherently require Fourier transform and inverse Fourier transform operations. Consequently, zero padding can be applied to spectrum signals DFT{*x*(*n*)} and DFT{*y*(*n*)} following the Fourier transform in cross-correlation-based time delay estimation to enhance the temporal resolution of the delay estimate. The computational complexity of interpolation can be significantly reduced by replacing the time-domain interpolation operations with equivalent frequency-domain zero padding. The Frequency-domain Zero-padded Cross-correlation Time Delay Estimation (FZC-TDE) algorithm operates on this fundamental principle, and its flowchart is shown in Figure 4.

## 3. Experimental Setup

**Figure 5:**
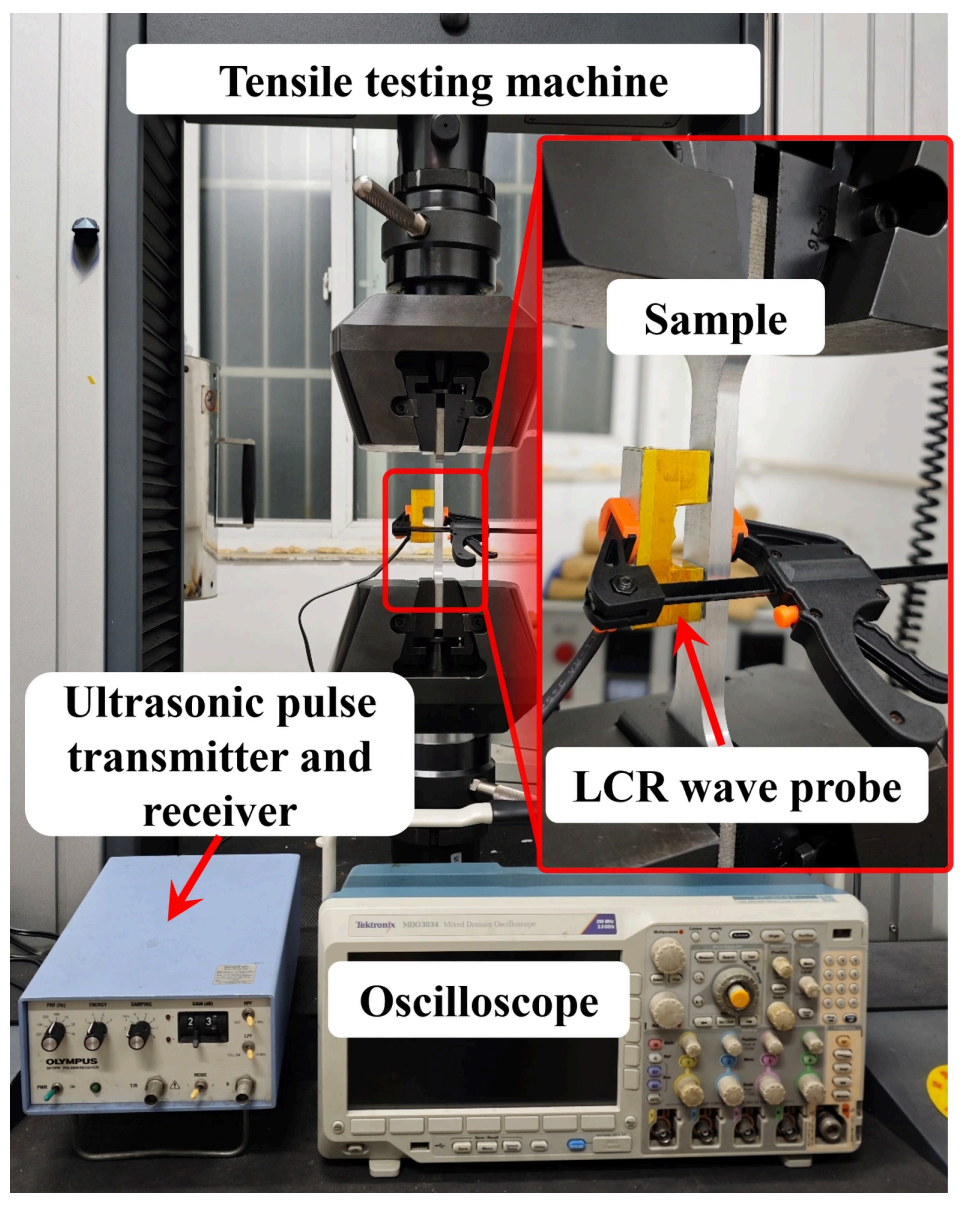
Photograph of the test platform.

Figure 5 shows a photograph of the test platform. A tensile machine (CMT5305, SASTest, Shenzhen, China) was used to apply stress of different values to tensile specimens, and an ultrasonic pulse emission receiver (5072PR, Olympus NDT, Waltham, Massachusetts, USA) was used to excite the LCR-wave transducer (UTS5M30L, STTech, Chengdu, China) to generate ultrasonic waves and receive the returned ultrasonic signals. We used an oscilloscope (MDO3034, Tektronix, Beaverton, Oregon, USA) for display and acquisition, the highest sampling rate of which was 2.5 GSPs, corresponding to a time resolution of 0.4 ns. A computer was used for the further processing and analysis of the LCR waves.

**Figure 6:**
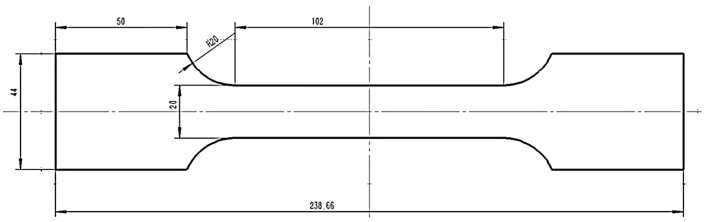
Tensile specimens for calibration experiments.

The tensile specimens used in the experiment consisted of a 6061-T4 aluminum alloy (provided by CHINALCO Inc., Tensile Yield Strength 145 MPa). For the calibration experiments, we used tensile specimens with thicknesses of 8 mm, with the remaining dimensions shown in Figure 6.

A step load of 0–120 MPa (with increments of 20 MPa) was applied to the tensile specimens with the tensile machine. A calibration test was carried out with the oscilloscope at a 2.5 GSPs sampling rate to obtain the stress coefficient of the specimen. Then, we set the sampling rate to 2.5 GSPs, 1 GSPs, 500 MSPs, 250 MSPs, 100 MSPs, 50 MSPs, 25 MSPs, and 10 MSPs to collect LCR-wave signals under different stress conditions. Finally, the differences between the measured stress values and the loading stress values of the stretching machine under different sampling-rate settings were compared. Moreover, in order to ensure the comparability of the data, all the settings were kept constant except for the sampling rate.

## 4. Results and Discussion

### 4.1. Effect of Sampling-Rate Variation

**Figure 7:**
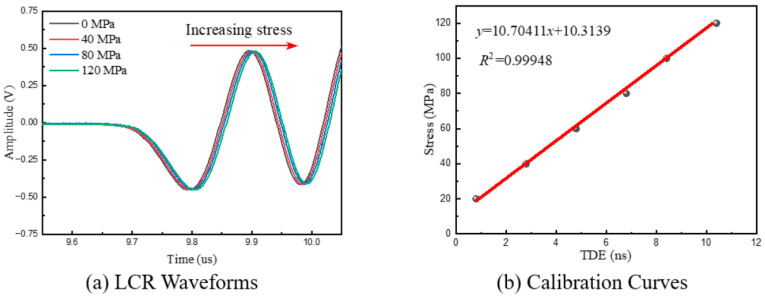
LCR waveforms and calibration curves under different stress conditions.

Figure 7a displays the LCR waves under varying stress levels at a sampling rate of 2.5 GS/s, revealing a progressive increase in wave propagation time with the increase in stress. Using the 0 MPa waveform as a reference, TDE was performed under various stress levels to establish the calibration curve, as shown in Figure 7b. The Pearson coefficient of the calibration curve is 0.99948, and the data show a strong linear relationship. This phenomenon is consistent with the above theoretical considerations regarding LCR-wave stress measurement, and the stress coefficient is K = 10.7041 MPa/ns.

**Figure 8:**
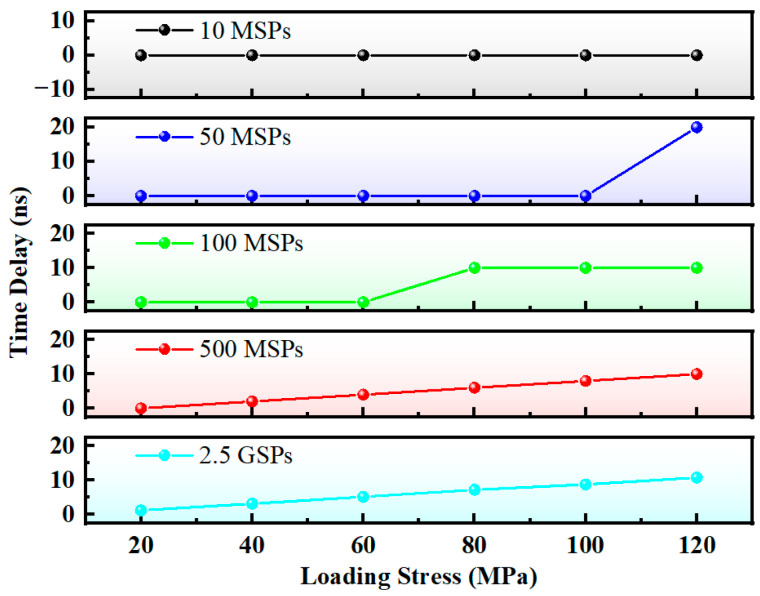
Time delay at different sampling rates (hardware-only sampling).

As exhibited in Figure 8, all measured time delay values become zero when the sampling rate is reduced to 25 MSPs. This is because under hardware-only sampling, the time resolution is determined by the hardware sampling rate; a rate of 25 MSPs corresponds to a time resolution of 40 ns. However, when the sample is stretched under a 120 MPa load, its delay only increases by approximately 10 ns, resulting in a time resolution that is insufficient to meet the measurement requirements.

Using a stress coefficient of *K* = 10.7041 MPa/ns, the time delay variation induced by a 1 MPa stress change can be estimated to be approximately 0.09342 ns. Therefore, to ensure a stress measurement resolution of 1 MPa, the corresponding time resolution must be at least 0.1 ns, which corresponds to a sampling rate of 10 GSPs. However, hardware capable of achieving a 10 GSPs sampling rate for hardware-only sampling is considerably expensive. Consequently, most existing schemes adopt a hardware sampling rate of over 1 GSPs and integrate a time-domain interpolation algorithm to achieve a temporal resolution of 0.1 ns.

**Figure 9:**
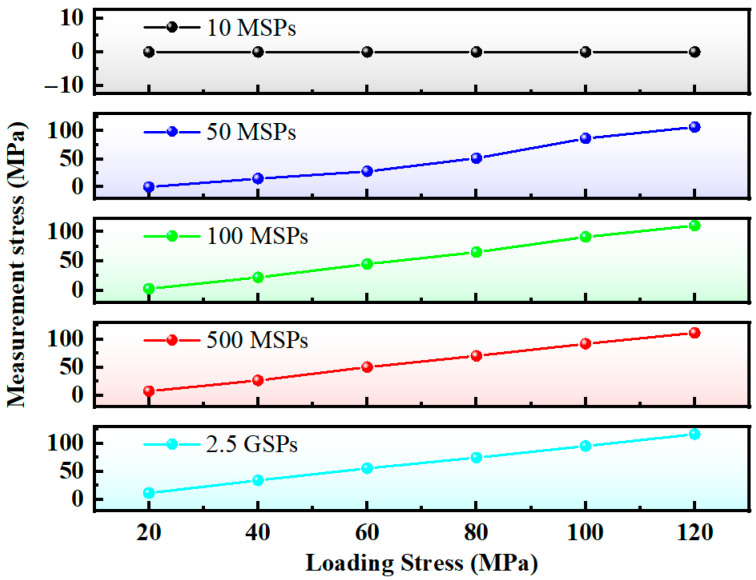
Measurement stress at different sampling rates (interpolated for 0.1 ns time resolution).

Waveform signals acquired at different sampling rates were processed with time-domain interpolation to achieve a 0.1 ns temporal resolution. Subsequently, the corresponding measured stress values were calculated, and the results are presented in Figure 9.

**Figure 10:**
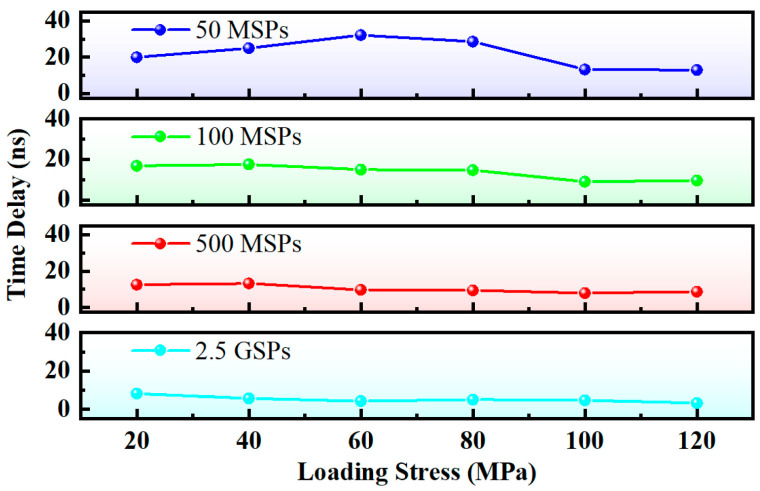
Measurement error at different sampling rates (interpolated for 0.1 ns time resolution).

As is evident from Table 1 and Figure 10, the measurement error exhibits an increasing trend with the decrease in the hardware sampling rate. When the latter is as low as 25 MSPs, effective time delay measurement remains unattainable even with the application of the interpolation algorithm.

**Figure 11:**
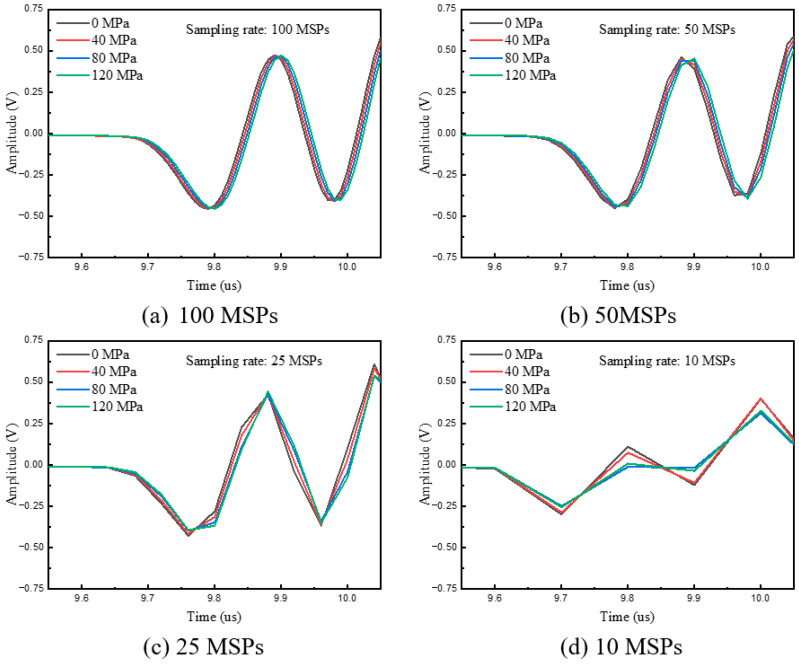
LCR waveforms under different sampling rates and stress levels.

As illustrated in Figure 11, the acquired waveform signals exhibit distortion when the sampling rate is below 50 MSPs. This is because the 50 MSPs sampling rate is only ten times the 5 MHz signal frequency, which is insufficient for accurate signal reconstruction, leading to distortion being further exacerbated, as shown by the line chart in Figure 11. This observation is consistent with the results shown in Table 1, which further indicates a significant increase in measurement error when the sampling rate drops below 50 MSPs. When the sampling rate is lower than 25 MSPs, the stress-induced delay variation cannot be effectively characterized—this is attributed to the insufficient number of sampling points within a single period.

The sensor utilized in this experiment had a center frequency of 5 MHz. According to the Nyquist theorem, a sampling rate of 10 MSps is sufficient to recover the original signal. However, the test results indicate that a sampling rate of at least 50 MSps is required to effectively assess the delay variation induced by stress. Furthermore, to meet the measurement requirements of ±10 MPa for high-stress segments (≥100 MPa), the sampling rate should not be lower than 100 MSps, which means that it should be no less than 20 times the signal frequency.

### 4.2. Comparison of Algorithms

Linear (Line) interpolation, Piecewise Cubic Hermite interpolation (PCH), and Cubic Spline interpolation (CS) were used to interpolate LCR-wave signals obtained at sampling rates of 1.25 GSps and 100 MSps; then, the cross-correlation (CC) TDE algorithm was used to calculate the time delay, and absolute measurement error values were obtained. Table 2 and Table 3 show that for different TDE algorithms, at a sampling rate of 100 MSps, the stress measurement error of the FZC-TDE algorithm is 10.3 MPa, while that of the other algorithms ranges from 7.4 MPa to 8.7 MPa. Moreover, as the sampling rate increases, the stress estimation error values calculated by different TDE algorithms tend to converge. Therefore, the results presented in these tables demonstrate that for different TDE algorithms, stress estimation accuracy remains unchanged at the same sampling rate.

**Figure 12:**
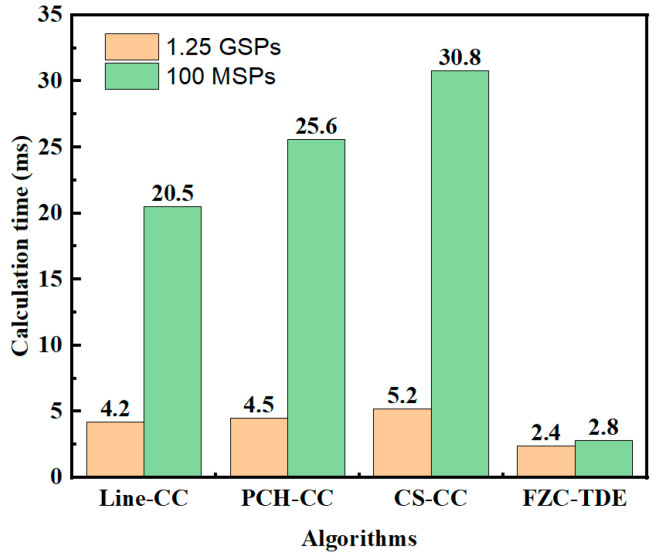
Comparison of calculation time of different algorithms.

Figure 12 shows the calculation time of different algorithms at different sampling rates; it can be seen that the sole difference among the algorithms is observed in their computational speed. When the sampling rate is 1.25 GSPs, the calculation time of the various time-domain interpolation algorithms is approximately 5 ms, and that of the FZC-TDE algorithm is around 2.4 ms. Upon reducing the sampling rate to 100 MSPs, the time-domain interpolation algorithms exhibit an increase in computation time to 20–30 ms, which is directly caused by the expansion of the oversampling factor from 8 to 100 times, and the computation time of the FZC-TDE algorithm is approximately 2.8 ms, without a substantial rise. Such stability not only confirms the reliable computational speed of the proposed algorithm but also emphasizes its significant advantage in terms of computational efficiency.

**Figure 13:**
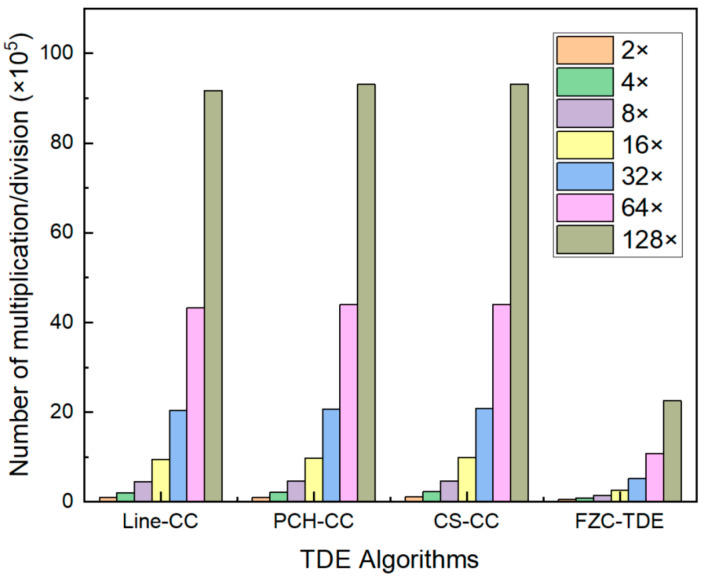
Number of multiplications and divisions required for different oversampling factors.

Table 4 shows the number of multiplications and divisions required for TDE with 128× oversampling on 1024 data points using different algorithms. It can be observed that the FZC-TDE algorithm requires only 2,258,944 multiplication and division operations, while the other algorithms require at least 9,175,040. This means that FZC-TDE uses only 25% of the operations required by the time-domain algorithms. Moreover, it can be observed that, for the time-domain TDE algorithm, the computational load of the Frequency domain TDE stage accounts for over 95% of all of its operations. For the FZC-TDE algorithm, however, since the signal processed during the FFT stage is the non-interpolated signal, which has only 1024 data points, the computational load for both the FFT and Multiply stages is significantly reduced compared to other algorithms, thereby effectively improving computational efficiency. Figure 13 presents the number of multiplications and divisions required for different oversampling factors. It is evident that compared to other algorithms, FZC-TDE exhibits slower growth in the required number of multiplications and divisions as the oversampling ratio increases, demonstrating superior stability.

## 5. Conclusions

This study presents a low-sampling-rate ultrasonic stress measurement method based on the FZC-TDE algorithm. We conduct tensile validation experiments at different sampling rates to determine the minimum hardware sampling-rate requirements for ultrasonic stress measurement. Additionally, FZC-TDE, a novel algorithm for real-time, high-resolution delay measurement at low sampling rates, is proposed. Specifically, the results from the tensile validation experiments indicate that when the sampling rate is less than 25 MSPs (even with the implementation of interpolation to ensure a 0.1 ns time resolution), temporal delay variations cannot be effectively characterized. To meet the measurement requirement of ±10 MPa, the sampling rate must be no less than 20 times the signal frequency. The comparative experiments examining different algorithms show that while various time-domain interpolation methods (Linear, PCH, and Cubic Spline) achieve comparable stress estimation accuracy at the same sampling rate, the FZC-TDE algorithm stands out significantly in terms of computational efficiency. At lower sampling rates (100 MSps), FZC-TDE maintains a stable computational time (~2.8 ms) with a stress error of 10.3 MPa, whereas the time-domain interpolation algorithms demonstrate increased computation times (20–30 ms) due to higher oversampling factors. Furthermore, FZC-TDE reduces the number of arithmetic operations by 75% (2.26 million vs. ≥9.18 million) for 128× oversampling, as it avoids processing interpolated signals in the frequency domain. Its computational load grows more slowly with oversampling ratios, highlighting its superior stability. Thus, FZC-TDE offers a compelling trade-off, balancing acceptable measurement accuracy with markedly improved efficiency, meaning that it is particularly suitable for real-time or resource-constrained applications. This study provides technical support for the development of micro–nano-sized processing equipment manufacturing and for the performance improvement and extended application of micro–nano-sized devices and systems.

## Figures and Tables

**Table 1:** Measurement error at different sampling rates (interpolated for 0.1 ns time resolution).

Sampling Rate	2.5 GSPs	1.25 GSPs	500 MSPs	250 MSPs	100 MSPs	50 MSPs	25 MSPs	10 MSPs
Loading Stress	Measurement Error (MPa)							
20 MPa	8.2	6.1	12.5	8.2	16.8	20.0	-	-
40 MPa	5.7	4.7	13.2	11.1	17.5	25.0	-	-
60 MPa	4.3	5.4	9.7	9.7	15.0	32.2	-	-
80 MPa	5.1	8.3	9.4	11.5	14.7	28.6	-	-
100 MPa	4.7	6.9	7.9	6.9	9.0	13.3	-	-
120 MPa	3.3	7.6	8.7	9.7	9.6	13.0	-	-

**Table 2:** Measurement stress of various algorithms (1.25 GSps).

Sampling Rate	Algorithm	Time-Domain Algorithms			FZC-TDE
		Line-CC	PCH-CC	CS-CC	
	Loading Stress	Absolute Measurement Error (MPa)			
1.25 GSps	−120 MPa	8.8	8.8	4.4	8.9
	−100 MPa	6.1	4.6	5.7	8.3
	−80 MPa	8.9	7.6	6.8	9.5
	−60 MPa	6.0	2.2	3.9	6.7
	60 MPa	4.7	5.1	1.7	5.4
	80 MPa	7.3	7.87.8	7.6	8.3
	100 MPa	4.8	6.9	6.1	6.9
	120 MPa	7.3	4.9	6.6	7.6
	Average	6.7	6.0	5.4	7.7

**Table 3:** Measurement stress of various algorithms (100 MSps).

Sampling Rate	Algorithm	Time-Domain Algorithms			FZC-TDE
		Line-CC	PCH-CC	CS-CC	
	Loading Stress	Absolute Measurement Error (MPa)			
100 MSps	−120 MPa	8.7	9.8	6.3	11.5
	−100 MPa	10.1	8.8	9.2	12.9
	−80 MPa	6.6	9.2	4.7	9.5
	−60 MPa	8.1	8.0	8.5	10.4
	60 MPa	9.0	9.4	9.1	9.7
	80 MPa	10.6	7.7	10.5	11.5
	100 MPa	6.6	4.1	4.1	6.9
	120 MPa	9.7	9.7	7.0	9.7
	Average	8.7	8.3	7.4	10.3

**Table 4:** Number of multiplications and divisions required for different algorithms.

Algorithm	Time Domain Interpolation	Frequency Domain TDE			Total
		FFT	Multiply	IFFT	
Line-CC	262,144	2,228,224	4,456,448	2,228,224	9,175,040
PCH-CC	398,336	2,228,224	4,456,448	2,228,224	9,311,232
CS-CC	405,504	2,228,224	4,456,448	2,228,224	9,318,400
FZC-TDE	/	10,240	20,480	2,228,224	2,258,944

## Data Availability

The original contributions presented in this study are included in the article. Further inquiries can be directed to the corresponding author.

## References

[1] Huff M. (2022). Review paper: Residual stresses in deposited thin-film material layers for micro- and nano-systems manufacturing. Micromachines.

[2] Kim T., Lee J. (2023). Fabrication and characterization of silicon-on-insulator wafers. Micro Nano Syst. Lett..

[3] Pachkawade V., Guha K. (2025). Micro- and Nano-Systems in 21st-Century: Designs, Developments, Applications and Perspective.

[4] Monine V.I., da Cruz Payão Filho J., Gonçalves Rios Alonso Munhoz M.C., de Assis J.T. (2025). Proposal of an alternative method for residual stress measurements in clad nickel alloy after shot peening. Measurement.

[5] Guo J., Fu H., Pan B., Kang R. (2021). Recent progress of residual stress measurement methods: A review. Chin. J. Aeronaut..

[6] Willemse P.F., Naughton B.P., Verbraak C.A. (1982). X-ray residual stress measurements on cold-drawn steel wire. Mater. Sci. Eng..

[7] Albertini G., Bruno G., Dunn B.D., Fiori F., Reimers W., Wright J.S. (1997). Comparative neutron and X-ray residual stress measurements on Al-2219 welded plate. Mater. Sci. Eng. A.

[8] Altpeter I., Dobmann G., Kröning M., Rabung M., Szielasko S. (2009). Micro-magnetic evaluation of micro residual stresses of the IInd and IIIrd order. NDT E Int..

[9] Chu Z., Li C., Liu J., Zhang J., Chen D., Wang L. (2024). Effect of anisotropy on residual stress measurement of 316L stainless steel by ultrasonic surface wave. Russ. J. Nondestruct. Test..

[10] Dive V., Lakade S. (2021). Recent research progress on residual stress measurement using non-destructive testing. Mater. Today Proc..

[11] Friedman V. (1994). A zero crossing algorithm for the estimation of the frequency of a single sinusoid in white noise. IEEE Trans. Signal Process..

[12] Yang F., Shi D., Lo L.-Y., Mao Q., Zhang J., Lam K.-H. (2023). Auto-diagnosis of time-of-flight for ultrasonic signal based on defect peaks tracking model. Remote Sens..

[13] Chen Z., Li Z. (2016). Robust precise time difference estimation based on digital zero-crossing detection algorithm. IEEE Trans. Instrum. Meas..

[14] Tan Y., Luo L., Li J., Zhang Y., Gao X., Peng J. (2022). Two-dimensional mean adaptive zero-crossing factor weighted ultrasound plane wave imaging. J. Nondestruct. Eval..

[15] Saxena H., Singh A., Rai J.N., Badoni M. (2022). PV integrated grid synchronization technique using modified SOGI-FLL and zero-crossing detector. Electr. Eng..

[16] Scarbrough K., Ahmed N., Carter G. (1980). An experimental comparison of the cross correlation and SCOT techniques for time delay estimation. Proceedings of the ICASSP ’80. IEEE International Conference on Acoustics, Speech, and Signal Processing.

[17] Sarwate D.V., Pursley M.B. (1980). Crosscorrelation properties of pseudorandom and related sequences. Proc. IEEE.

[18] Knapp C., Carter G. (1976). The generalized correlation method for estimation of time delay. IEEE Trans. Acoust. Speech Signal Process..

[19] Liu Q., Zhou B., Zhao R., Dai M., Wang B., Wang Y. Development of acoustic thermometer and velocimeter with high temporal resolution and noise suppression capability. Proceedings of the 2022 11th International Conference on Communications, Circuits and Systems (ICCCAS).

[20] Zhang C., Shen S., Huang H., Wang L. (2021). Estimation of the vehicle speed using cross-correlation algorithms and MEMS wireless sensors. Sensors.

[21] Guschina O. (2021). Refining Time Delay Estimate of Complex Signal Using Polynomial Interpolation in Time Domain. Proceedings of the 2021 Systems of Signals Generating and Processing in the Field of on Board Communications.

[22] Sun S., Li S., Lin L., Yuan Y., Li M. (2019). A novel signal processing method based on cross-correlation and interpolation for ToF measurement. Proceedings of the 2019 IEEE 4th International Conference on Signal and Image Processing (ICSIP).

[23] Chen B., Luo C., Xia L., Xu L., Yan G., Qiu F., Gou G. (2024). Research on the measurement technology for pretension stress on small-sized bolts based on the piezoelectric ultrasonic resonance method. Materials.

[24] Chen B., Chen J., Qiu F., Luo C., Gou G. (2025). Ultrasonic stress measurement in thin-walled structures via shear wave birefringence. Meas. Sci. Technol..

[25] Kim H., Kim T., Morrow D., Jiang X. (2020). Stress Measurement of a Pressurized Vessel Using Ultrasonic Subsurface Longitudinal Wave with 1–3 Composite Transducers. IEEE Trans. Ultrason. Ferroelectr. Freq. Control.

[26] Egle D.M., Bray D.E. (1976). Measurement of acoustoelastic and third-order elastic constants for rail steel. J. Acoust. Soc. Am..

[27] Hanus R. (2019). Time delay estimation of random signals using cross-correlation with hilbert transform. Measurement.

[28] Zhang S., Shen G., An L. (2019). Online monitoring of furnace exit gas temperature in power plants. Appl. Therm. Eng..

[29] Tu Y.-Q., Shen Y.-L. (2017). Phase correction autocorrelation-based frequency estimation method for sinusoidal signal. Signal Process..

[30] Rockwood A.L., Crockett D.K., Oliphant J.R., Elenitoba-Johnson K.S.J. (2005). Sequence alignment by cross-correlation. J. Biomol. Tech..

[31] Kong L., Zhang L., Guo H., Zhao N., Xu X. (2024). Time delay study of ultrasonic gas flowmeters based on VMD–hilbert spectrum and cross-correlation. Sensors.

[32] Gong Z. (2024). Fast signal interpolation through zero-padding and FFT/IFFT. arXiv.

[33] Nelson M.A., Rooney B.D., Dinwiddie D.R., Brunson G.S. (2003). Analysis of digital timing methods with BaF_2_ scintillators. Nucl. Instrum. Methods Phys. Res. Sect. A Accel. Spectrometers Detect. Assoc. Equip..

[34] de Lima D.V., Miranda R.K., Galdo G.D. High resolution time-delay estimation via direction of arrival estimation and khatri-rao factorization for multipath mitigation. Proceedings of the WSA 2017, 21th International ITG Workshop on Smart Antennas.

[35] Aliaga R.J. (2017). Real-time estimation of zero crossings of sampled signals for timing using cubic spline interpolation. IEEE Trans. Nucl. Sci..

